# Book Review: The Story of Catch: The Story of Lancashire Catch-as-Catch-Can Wrestling

**DOI:** 10.3389/fpsyg.2020.02003

**Published:** 2020-08-26

**Authors:** Kazimierz Waluch

**Affiliations:** ^1^Instytut Rozwoju Sportu i Edukacji, Warszawa, Poland; ^2^Management Department, Pawel Wlodkowic University College in Płock, Płock, Poland

**Keywords:** traditional sports, roots of wrestling, intangible heritage, sport, tradition, culture, cultural identity

I present a review on the book “The Story of Catch” by Ruslan C. Pashayev a wrestling history enthusiast from Columbus, Ohio, United States who is also an expert-member of the Traditional Sports Team of the Instytut Rozwoju Sportu i Edukacji (the Institute of Sport Development and Education), Warsaw, Poland. Meticulous research and attention to detail have made Ruslan C. Pashayev one of the foremost authorities on the history of Western European Catch-Hold traditional styles of wrestling (folk wrestling styles), particularly the Lancashire Catch-as-Catch-Can style.

“The Story of Catch” is the only major book ever to cover this subject or the time period that began a variation of professional wrestling which is known around the world as “Lancashire Catch Wrestling” or simply “Catch.” Besides that “The Story of Catch” covers the history of various Western European traditional catch-hold wrestling styles which predated and “fathered” Lancashire catch-as-catch-can as well as tracing the origin and explaining the evolution of modern International Olympic styles of wrestling such as Graeco-Roman and Freestyle. The purpose of his work was to establish the truth about the origin of Lancashire catch wrestling and explain the evolution of this cultural phenomenon.

Seven years of research in various libraries, local studies, historical societies, archives, registration and record offices, and museums in Great Britain in particular those located in the towns of East Lancashire (Wigan, Bolton, Bury, Middleton, Rochdale, Oldham, Ashton, Blackburn, and Burnley) and in West Yorkshire, as well as in the British Library, American Library of Congress, and many others. The memoirs of individuals, historical books, documents, newspapers, and magazines all have played a part in the overall success of the studies that led to this work.

This book presents a pivotal study in restoring the history of Lancashire catch-as-catch-can wrestling. Ruslan C. Pashayev's research clearly is both revolutionary and eye-opening. The author has done an amazing work of research in tracking down and documenting the true origins of catch-as-catch-can wrestling. He cuts through the myths and legends that have evolved around this wrestling style over the decades and presents irrefutable evidence as to its introduction to the Lancashire County of England and its amazing growth in the mid and late nineteenth century. By analyzing thoroughly historical documents, including newspapers of the time, the author has successfully challenged much of the traditionally perceived history of the sport. His inquiries resulted in an absorbing revelation of the foundations and development of a sport that was destined to become a social phenomenon of the twenty-first century. His investigations have unveiled the ethnic and geographical origins of the Lancashire Catch-as-Catch-Can style, the wrestling styles that preceded Catch in the region of East Lancashire, and the emergence of a professional category in this sport. Like a modern day detective Ruslan C. Pashayev has pieced together the forensic evidence to weave an absorbing chronicle of Lancashire wrestling's fascinating history. Besides that it is written clearly, with attention to detail and humor that only one with a deep understanding of the wrestling sport could even appreciate.

The book starts with the author giving very detailed information and analyses of the various traditional wrestling styles (both catch-hold and fixed-hold styles of wrestling) which were historically practiced in different parts of England in particular in the areas of East Lancashire and West Yorkshire. Then author explains the history of the term of Catch-as-catch-can (catch/take a hold of someone as one pleases) wrestling in England throughout the centuries and resumes by providing a great account of two kinds of catch wrestling styles which were around in the early 1800s, the London Catch-as-catch-can and Lancashire Catch-as-catch-can styles of wrestling.

Among the revolutionary discoveries made by the author was the introduction of the up and down catch-as-catch-can wrestling by the immigrants (textile workers) from Continental Europe (originally from Flanders) in East Lancashire and West Yorkshire in the fourteen to seventeen centuries (since 1560s the Protestant wave of immigrants which besides Flemish also included Germans and French Huguenots) and its transformation into a professional Lancashire up and down fighting and establishment its major centers in the towns of East Lancashire (Pre-1800s). Tracing the roots of Lancashire catch-as-catch-can wrestling to the “Flemish weavers” the immigrants from the Medieval County of Flanders who brought their free-for-all traditional style of wrestling called Stoeijen to East Lancashire was instrumental in explaining two major features of Lancashire catch wrestling (catch hold of any part of the body above and under the waist and ground wrestling) which significantly differed from those of the traditional English styles of wrestling and weren't found anywhere else in England outside the areas of East Lancashire and West Yorkshire. Those two features of Lancashire catch wrestling previously remained without any explanation for so long until the author of this book successfully resolved this major historical paradox.

Further in the book the author talks about the abolition of illegal pro Lancashire up and down fighting and introduction of the modern pro catch wrestling in East Lancashire which happened in the 1820s. The information about the establishment of the Rules of the Game which occurred in the 1840s-50s (the time period which produced the first generation of Lancashire pro wrestling superstars) was uniquely interesting. Arguably the most important part of the book from the historical point of view is the one which talks about the Golden Era of Catch Wrestling, the 1860s. In this the section of the book the author provides previously unknown detailed information about the earliest pro wrestling promotions (championship titles), the great champions and championship lineages of the most glorious epoch of catch. The Author fully and completely explains the rules according to which the pro wrestling business was operated in East Lancashire and how it affected and shaped the modern day pro wrestling.

In the second part of the book the author highlights pro wrestling during the so-called Era of Claimants, the epoch which followed the heyday of Lancashire wrestling and the immigration of Lancashire Superstars to America in the 1870s-1900s.

In the very end of the book the author is giving a descriptive analyses of the early 1900s situation in pro catch wrestling in Britain during the era called the Wrestling Boom or the music halls era of catch (1899- Pre-WW1) as well as he speaks about the destiny of catch wrestling in twentieth century (Britain, North America, and Continental Europe) and prophesizes about it's future.

The research presented in this book was rated as being of high historical value by the foremost American (such as Mark Hewitt and Steve Yohe) and British (such as Alan Bamber and Ron Harrison) wrestling historians. “The Story of Catch” was very well received all around the world and is now promoted on the various wrestling history studies websites such as Traditional Sports (traditionalsports.org), Wrestling Heritage of UK (wrestlingheritage.co.uk), Pro Wrestling Historical Society (prowrestlinghistoricalsociety.com), and Wrestling Titles History (wrestling-titles.com).

“The Story of Catch” also presents numerous historical photos, captions, and illustrations, some of which have never been seen before. Whether one is a wrestling fan, wrestling history scholar, wrestling and MMA enthusiast, or practitioner the research of this indefatigable historian and his “The Story of Catch” are a must read.

## Author Contributions

The author confirms being the sole contributor of this work and has approved it for publication.

## Conflict of Interest

The author declares that the research was conducted in the absence of any commercial or financial relationships that could be construed as a potential conflict of interest.

